# Kinetics of ion release from a conventional glass-ionomer cement

**DOI:** 10.1007/s10856-021-06501-1

**Published:** 2021-03-16

**Authors:** John W. Nicholson, Nichola J. Coleman, Sharanbir K. Sidhu

**Affiliations:** 1 Dental Physical Sciences Unit, Queen Mary University of London, Mile End Road, London, E1 4NS UK; 2 Bluefield Centre for Biomaterials, 67-68 Hatton Garden, London, EC1N 8JY UK; 3 School of Science, University of Greenwich, Medway Campus, Chatham, Kent ME4 4TB UK; 4 Centre for Oral Bioengineering, Institute of Dentistry, Queen Mary University of London, Turner Street, London, E1 2AD UK

## Abstract

Release kinetics for sodium, silicon, aluminium, calcium and phosphorus from conventional glass-ionomer dental cement has been studied in neutral and acid conditions. Specimens (6 mm height × 4 mm diameter) were made from AquaCem (Dentsply, Konstanz, Germany), 6 per experiment. They were matured (37 °C, 1 h), then placed in 5 cm^3^ storage solution at 20–22 °C. In the first experiment, deionised water, changed daily for 28 days, was used. In the second, deionised water, changed monthly for 21 months, was used. In the third, lactic acid (20 mmol dm^−3^, pH: 2.7 ± 0.1), changed monthly for 21 months was used. After storage each solution was analyzed by inductively coupled plasma-optical emission spectroscopy (ICP-OES). Results showed that in neutral conditions, no calcium was released, but in acid, significant amounts were released. The other elements (Na, Al, Si and P) were released in neutral as well as acid conditions, with greater amounts in acid. More frequent changes of water gave greater release. In neutral conditions, release over 21 months followed the equation: [*E*]_c_ = [*E*]_1_*t*/(*t* + *t*_½_) + *β*√*t* ([*E*]_c_ is the cumulative release of the element). In acid conditions, this became: [*E*]_c_ = [*E*]_1_*t*/(*t* + *t*_½_) + *αt*. Hence release of all elements was shown to occur in two steps, a rapid initial one (half-life: 12–18 h) and a longer second one. In neutral conditions, the longer step involves diffusion; in acid it involves erosion. These patterns influence the material’s bioactivity.

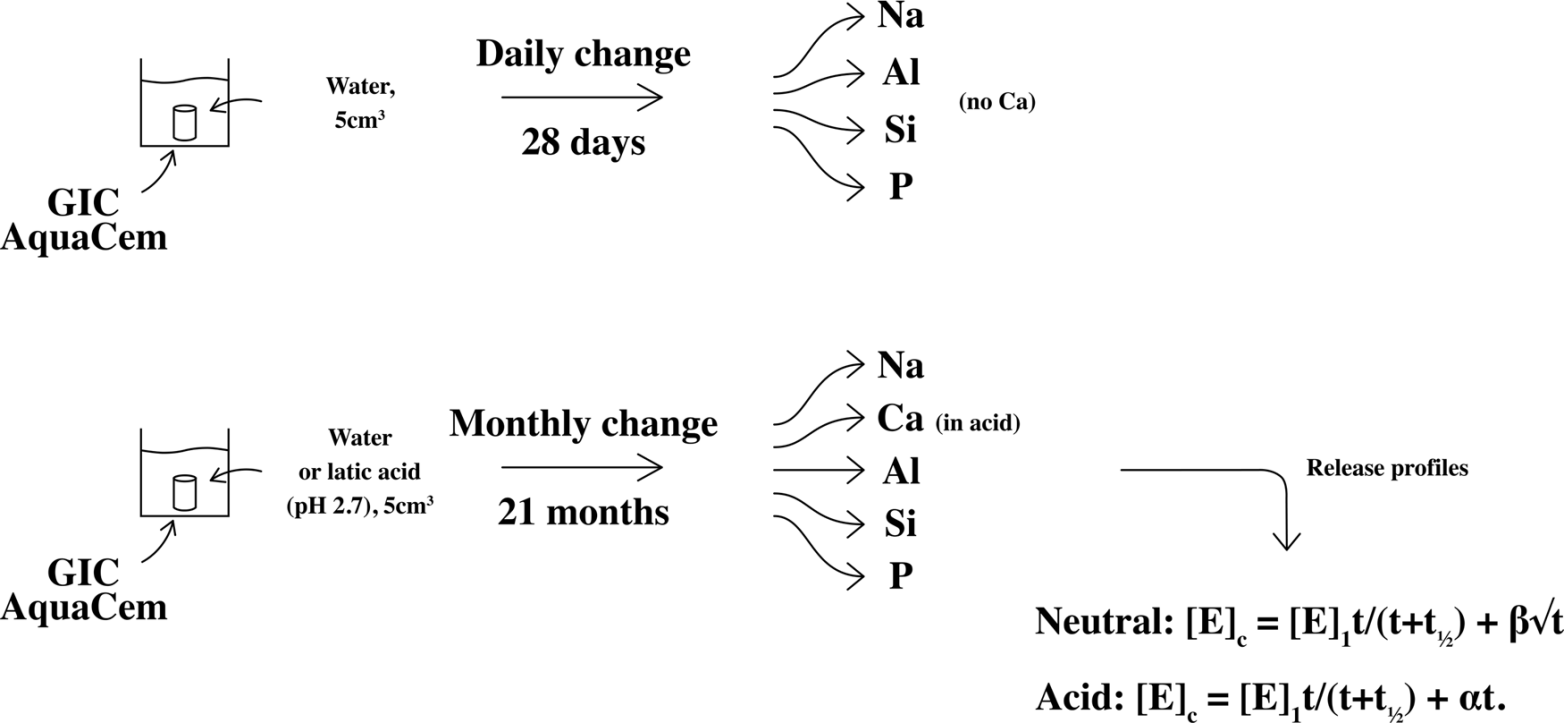

## Introduction

Glass-ionomer cements are widely used in contemporary clinical dentistry [1, 2]. Applications include as liners and bases, full restorative materials, luting cements, pit and fissure sealants, and orthodontic adhesives [1, 2]. In their conventional version, they are prepared by reaction of special basic glass powders with aqueous solutions of polymeric acids, such as poly(acrylic acid), acrylic acid–maleic acid copolymer, or the copolymer of 2-methylene butanedioic acid with propenoic acid [3]. They are also available as resin-modified materials, where there is an additional monomer, usually 2-hydroxyethyl methacrylate. There is also an appropriate chemical initiator system to promote free-radical polymerization of the monomer component [2].

One of the important properties of glass-ionomers of both types (conventional and resin-modified) is the ability to release fluoride. This property has been widely studied [4, 5], and much is known about it. In principle, it is beneficial, because fluoride at low concentrations helps promote remineralization of the hydroxyapatite component of the tooth, and thereby to reverse the damage caused by caries, to some extent [6, 7]. However, whether glass-ionomers release sufficient fluoride to do this to any significant extent has not been established [8, 9].

The kinetics of fluoride release has been studied under both neutral and acidic conditions [10, 11]. For both types of glass-ionomer, in neutral conditions, the cumulative release [*F*]_c_ is given by the equation:

1 $$\left[ {F} \right]_{\rm{c}} = \left[ {F} \right]_{1}{t}/\left( {{t} + {t_{1/2}}} \right) + \beta \surd {t}$$

This shows that release occurs in two steps. The first of these is sometimes referred to as early washout, and is a first-order dissolution stage that ceases after some time. It is accounted for by the first term in the equation. The second stage is a longer term, slower process that is diffusion based, as shown by the square root of time dependency of the second term in the equation [12]. This process is held to be responsible for the ability of glass-ionomer cements to release fluoride for extended periods of time [10].

In this equation, [*F*]_1_ is the amount of fluoride released by stage 1, and it can be determined from a plot of fluoride release against square root of time. The slope of such a plot is the term *β* and the intercept approximates to [*F*]1. The term *t*_½_ can be calculated once the [*F*]_1_ term has been estimated. In this way, all of the terms in the equation can be found, and the extent to which the calculated value of [*F*]_c_ fits the experimental values determined. When this is done, there is excellent agreement between calculated and experimental values [10].

Under acidic conditions, there is typically greater fluoride release [13, 14], and the kinetics changes so that the equation describing release becomes:

2 $$\left[ {F} \right]_{\rm{c}} = \left[ {F} \right]_{1}{t}/\left( {{t} + {t_{1/2}}} \right) + \alpha {t}$$

This suggests that release still occurs in two steps, but that the acidic conditions alter the fundamental nature of the second, longer-term release process. Instead of being diffusion based, it becomes a simple linear function of time, indicating that it is due to slow dissolution of the cement in the low-pH storage medium. The terms in this equation can be estimated by similar means to those in Eq. 1, and under acidic conditions, the agreement between calculated and experimental values is best when the latter equation is used [10, 11].

Glass-ionomer cements of both types release other ions in addition to fluoride. In particular, in neutral conditions, they release sodium, aluminium and silicate species [15, 16]. Depending on the formulation, they may also release phosphate species, and these have been shown by ion chromatography to be mainly orthophosphate, PO_4_^3−^ together with another phosphate ion that has been tentatively identified as mono-fluorophosphate, PO_3_F^2−^ [17]. They do not release either calcium or strontium ions at neutral pH. By contrast, under acidic conditions, they release one or other of these ions in reasonable amounts, and they also release increased amounts of the other species (Na^+^, Al^3+^, silicates, phosphates) compared with neutral conditions [15, 16].

The ability to release ions is the basis of the bioactivity of glass-ionomers. The term “bioactivity” has mainly been applied to the speciality glasses developed originally by Hench and his co-workers in the years from 1969, the so-called “bioactive glasses” [18]. The term has been defined as the ability of a material to form a true mechanically compliant bond with the host biological tissue without the formation of fibrous capsule [19]. Conventional glass-ionomers have been shown to do this with tooth surfaces, especially dentine [20, 21]. In contact with such surfaces, conventional glass-ionomers promote a slow chemical reaction that results in a distinct interfacial zone that is mechanically strong and capable of resisting chemical attack by acids [20]. Elemental analysis has shown that, when formed from a strontium-based cement, it contains both calcium and strontium. This indicates that its formation involves diffusion of calcium from the tooth and strontium from the cement into this interfacial structure. The presence of this structure causes the cement to adhere strongly to the tooth.

The bioactivity of glass-ionomers is a topic of growing importance, and recent studies have attempted to improve this feature by the addition of ion-releasing components, such as bioactive glass [22] or calcium silicates [23] to the cement. There has been some question in the literature as to whether glass-ionomers are inherently bioactive [23], and whether they need additives to become so. However, on the basis of Hench’s definition, and the observations of ion-exchange bonding [20, 21] it seems they definitely are bioactive [5], even if this property can be improved by the use of additives or by formulating the cement from alternative acid and glass components [24].

The related materials, resin-modified glass-ionomers have also been found to adhere well to the tissues of the tooth [25, 26]. Images of bonded cements have been published, but only for relatively immature specimens [25]. There are no images for specimens of resin-modified glass-ionomer cements that have been bonded to teeth for several years, so no comparable evidence for the formation of an ion-exchange layer with these materials.

The release of mineralizing ions from conventional glass-ionomers is therefore important. However, so far, there have been no reports on the kinetics of the release process. As we have seen, the total release of these ions is greater under acidic conditions than neutral ones, and the release profile changes to include either calcium or strontium, depending on the composition of the glass used. However, to date no information has been published on the release profiles of these ions.

The current study seeks to remedy this for a conventional glass-ionomer cement. In this study, release profiles for Na^+^, Ca^2+^, Al^3+^, silicates and phosphates have been determined for up to 21 months in both neutral and acidic conditions. Curve fitting processes similar to those used for fluoride have been applied to the data, using the following modified versions of the release equations:

3 $$\left[ {E} \right]_{\rm{c}} = \left[ {E} \right]_{1}{t}/\left( {{t} + {t_{1/2}}} \right) + \beta \surd {t}$$

4 $$\left[ {E} \right]_{\rm{c}} = \left[ {E} \right]_{1}{t}/\left( {{t} + {t_{1/2}}} \right) + \alpha {t}$$

where [*E*]_c_ is the cumulative release of element *E* at time *t* months, and [*E*]_1_ the release of element *E* due to step 1.

In addition, a preliminary short-term (28 days) experiment was carried out, designed to mimic the earlier study of fluoride release [10], where fluoride release was measured at daily intervals, at least initially. This allowed the values of *t*_½_ under such conditions to be determined for each element and compared with that for fluoride.

Finally, for longer-term (21 months) release profiles, curve fitting has been used to determine which of the two possible kinetic equations best describes the release of each ion under both types of storage condition. The aim was to compare the release profiles of all of the species released with those of fluoride, whose release profiles in both neutral and acidic conditions have been reported previously [10, 11].

## Materials and methods

The experimental work was carried out using the conventional glass-ionomer brand AquaCem (Dentsply, Konstanz, Germany). This is a water-activated material, and specimens were prepared by mixing in the mass ratio 3.3:1 on a mixing pad using a metal spatula, for approximately 15 s until a smooth consistency was achieved. Individually mixed samples of cement were placed in metal moulds (6 mm height × 4 mm diameter), and stored in an incubator at 37 °C for 1 h before being removed from the mould and placed in 5 cm^3^ volumes of deionised water in clean plastic 25 cm^3^ centrifuge tubes. Sets of six specimens were prepared for each of the experiments performed.

Two series of experiments were carried out. The first was aimed at determining the value of *t*_½_ for each element with daily replenishment of the storage water, in order to compare the values with those obtained previously for fluoride from commercial glass-ionomer cements [10]. In this series of experiments, individual specimens of glass-ionomer cement were placed in 5 cm^3^ volumes of deionised water in clean plastic 25 cm^3^ centrifuge tubes and stored at room temperature (20–22 °C) for 1 day, after which they were removed and placed in fresh 5 cm^3^ volumes of deionised water. This was repeated for 28 days, and the samples of storage water were then analyzed using inductively coupled plasma-optical emission spectroscopy (ICP-OES). From this, individual graphs of cumulative ion release were plotted, and the data used to determine the half-life for step 1 in the kinetic equations.

In the second series of experiments, specimens were stored for monthly intervals, being transferred into fresh 5 cm^3^ volumes of de-ionized water each month for 21 months. Again, the storage temperature was in the range 20–22 °C throughout these experiments. The samples of storage water were analyzed by ICP-OES and the values used to determine which of the two kinetic equations best fitted the experimental data.

In a parallel long-term study, specimens were prepared and stored in 5 cm^3^ volumes of lactic acid solution (concentration: 20 mmol dm^−3^, pH: 2.7 ± 0.1). As for specimens stored in deionised water, a set of six specimens was stored for 1-month intervals before being transferred to fresh volumes of lactic acid solution each month. This was continued for 21 months. At the end of this time, the values were used to determine which of the two kinetic equations best fitted the experimental data. In addition, the total release of each ion/species into acid solution was compared with that for release into water.

All solutions were analyzed by ICP-OES. This was performed on a Perkin Elmer ICP-OES Optima 4300 DV system (Beaconsfield, Buckinghamshire, UK). The five analytes of interest (Na, Ca, Al, Si and P) were located by aspirating 10 ppm of mixed aqueous solutions into the plasma in order to obtain the signal and lock into their position by reference to mercury using the Hg lamp and auto-calibrating at 20-min intervals to compensate for small variations in optical position. Sample solutions were prepared and stored in pre-cleaned plastic vessels. All calibration measurements were performed in triplicate and analyses were performed in duplicate and averaged to give 12 measurements per time interval and storage time combination.

Limits of detection and quantification were determined experimentally for each of the analytes in both media, using the statistical approach described by Vogelgesang and Hadrich [27].

Once data had been collected for release of the five chemical species (Na, Ca, Al, Si and P) over 21 months, the cumulative total release of each was determined by adding the monthly totals together. Curve fitting using non-linear regression analysis was performed on each set of monthly release data using the modified Eqs. 3 and 4. This used the software SPSS 14.0 for Windows to perform curve-fitting analysis, and employed the Levenberg–Marquardt algorithm to find the coefficients that gave the best fit between the experimental data and the equations. The adequacy of these equations was determined, as in previous studies, based on the correlation coefficient *R*.

In addition, for the release data for ion release in neutral and acidic conditions, normality was checked using the Shapiro–Wilk test and the significance of any differences was tested using the Student *t* test.

## Results

The results for the determinations of limits of detection and quantification are shown in Table 1. They show that the ICP-OES was sufficiently sensitive to detect and quantify all the analytes of interest in this study. Ion release data were shown to be normally distributed (*p* > 0.05) according to the Shapiro–Wilk test.

**Table 1:** Limits of detection (LoD) and quantification (LoQ) for analytes released from glass-ionomer cement

Analyte	LoD in water/ppb	LoQ in water/ppb	LoD in lactic acid/ppb	LoQ in lactic acid/ppb
Na	2.52	8.40	2.05	6.83
Ca	2.57	8.57	2.33	7.77
Al	3.94	13.14	3.40	11.34
Si	6.91	23.03	5.37	17.90
P	6.77	22.55	5.65	18.85

**Fig. 1:**
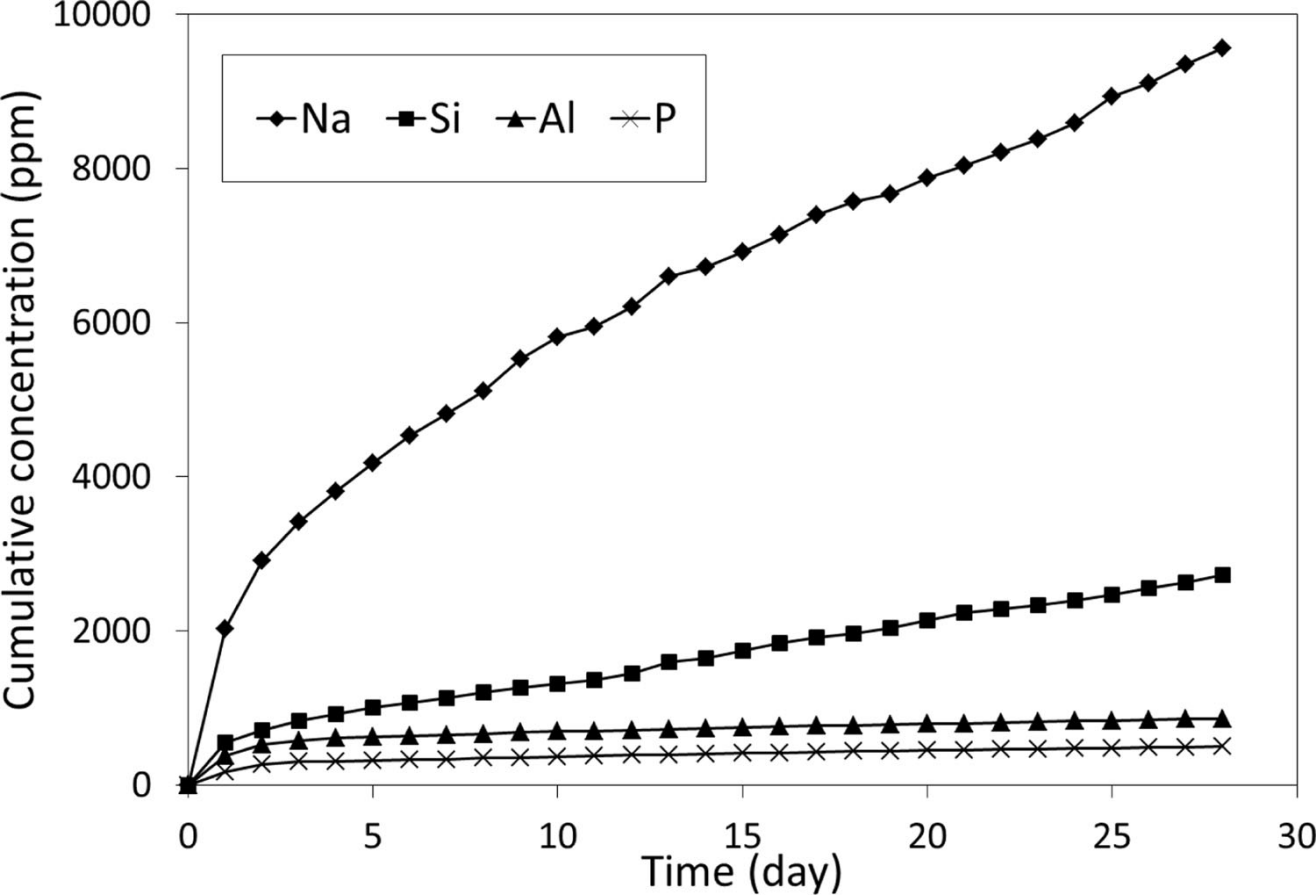
Twenty-eight-day cumulative release of Na, Si, Al and P under neutral conditions

**Fig. 2:**
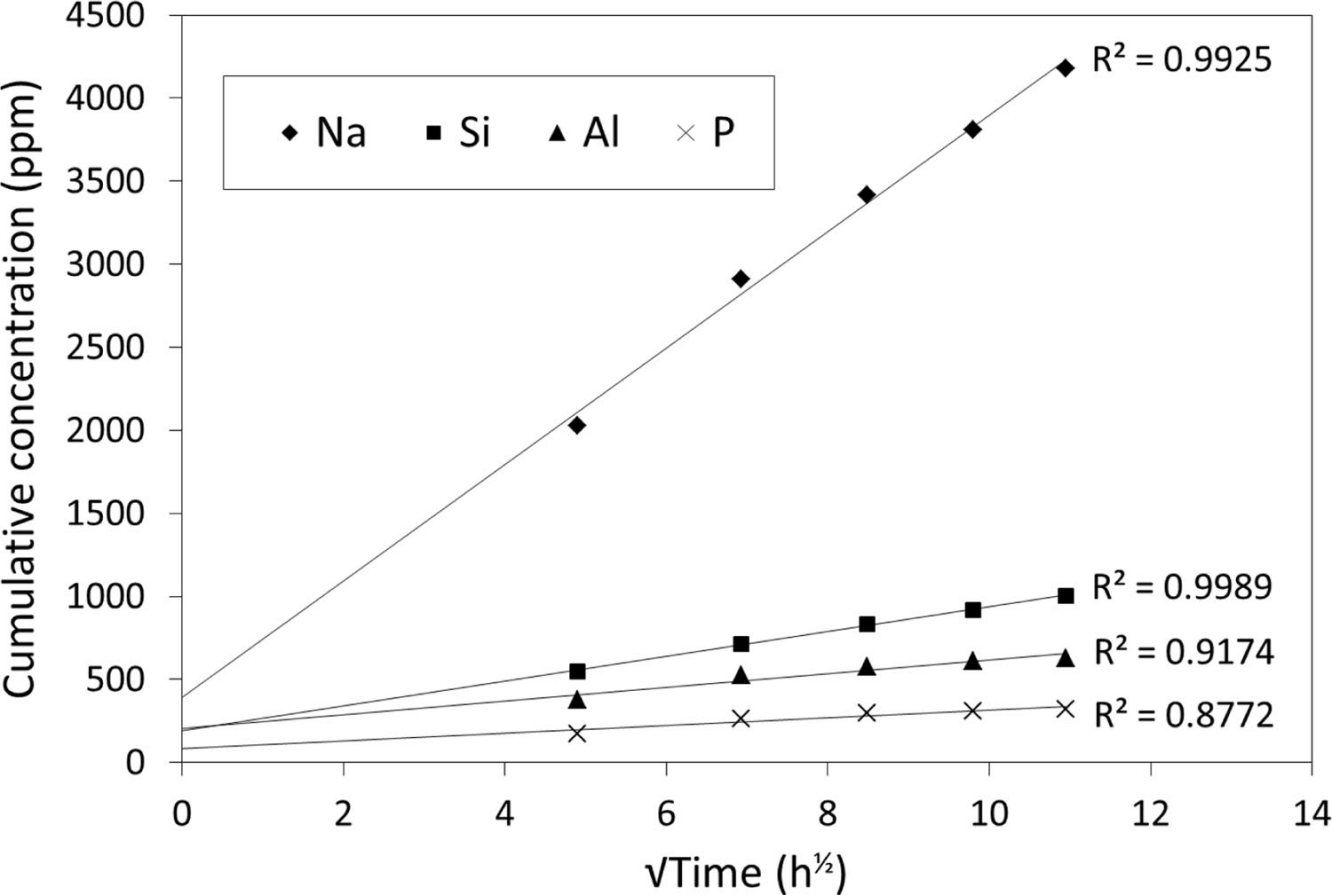
Plots of concentration versus the square root of time for the dissolution of Na, Si, Al and P under neutral conditions

Storage in neutral conditions was found to give negligible amounts of calcium for this cement, but reasonable amounts of sodium, aluminium and silicon, and also a measurable amount of phosphorus (as shown in Fig. 1). Figure 2 shows the plots of concentration versus the square root of time for the release of these elements. In each case, the data gave straight lines. For short-term release (28 days) two key values, namely *t*_½_ and *β*, could be determined for these elements, and these are shown in Table 2. The values of *β* were obtained from the slopes of the graph in Fig. 2. Because these lines are plotted from data collected at relatively long time periods, where *t* ≫ *t*_½_, the term [*E*]_1_*t*/(*t* + *t*½) becomes effectively [*E*]_1_.

**Table 2:** Release parameters for short-term (28 days) release under neutral conditions for Na, Si, Al and P

Element	[*E*]_1_ (ppm)	*t*_½_ (h)	*β* (ppm h^−½^)
Na	390	14.1	325.0
Si	191	12.7	69.2
Al	206	18.1	16.1
P	84	18.6	3.5

In all cases, the value of *t*_½_ is estimated as less than 24 h. The value of *β* has been described as a measure of the driving force for fluoride ion release in previous studies [10], and that can be considered the case for the ions reported in Table 2.

**Fig. 3:**
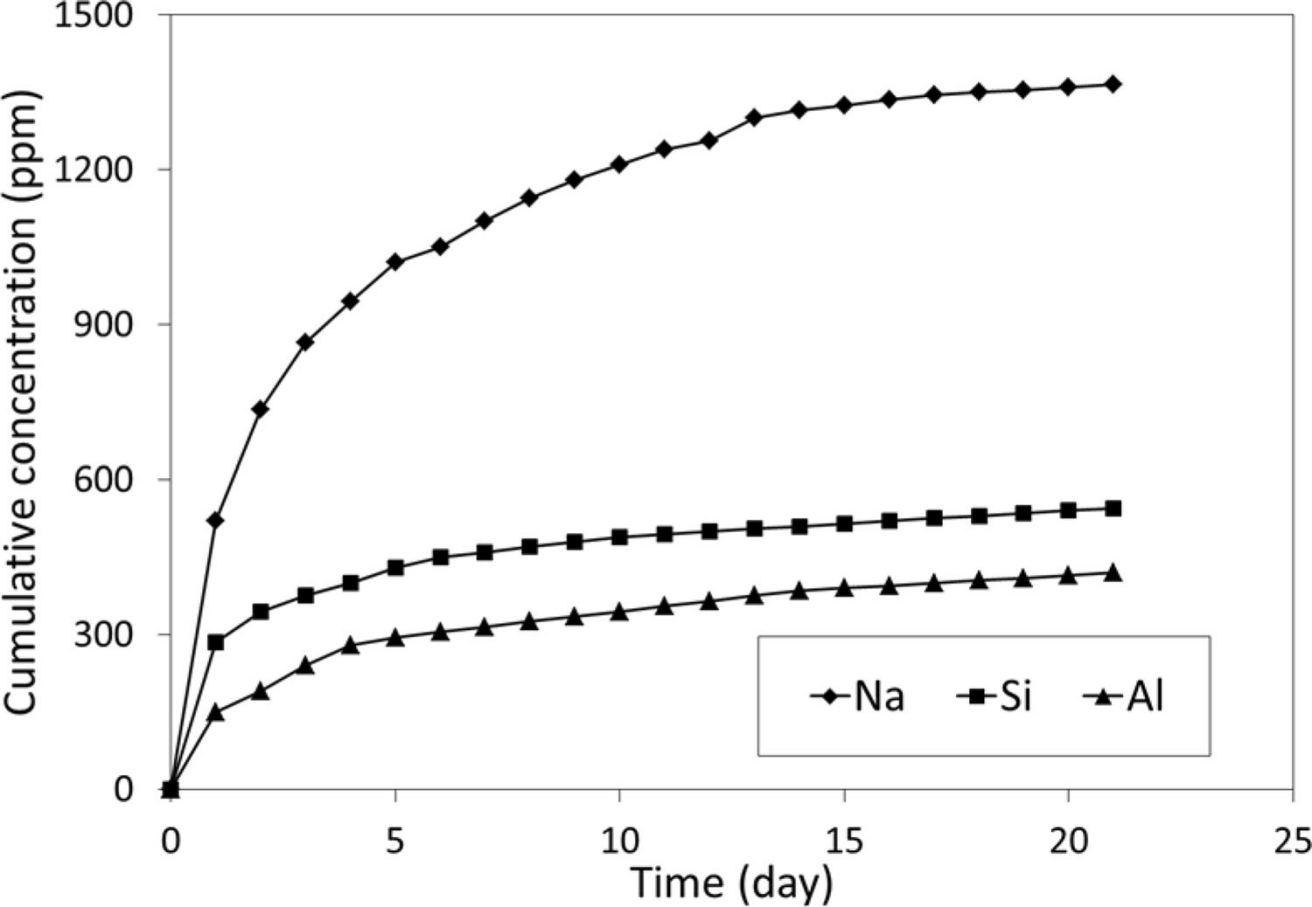
Twenty-one-month cumulative release of Na, Si and Al under neutral conditions

**Fig. 4:**
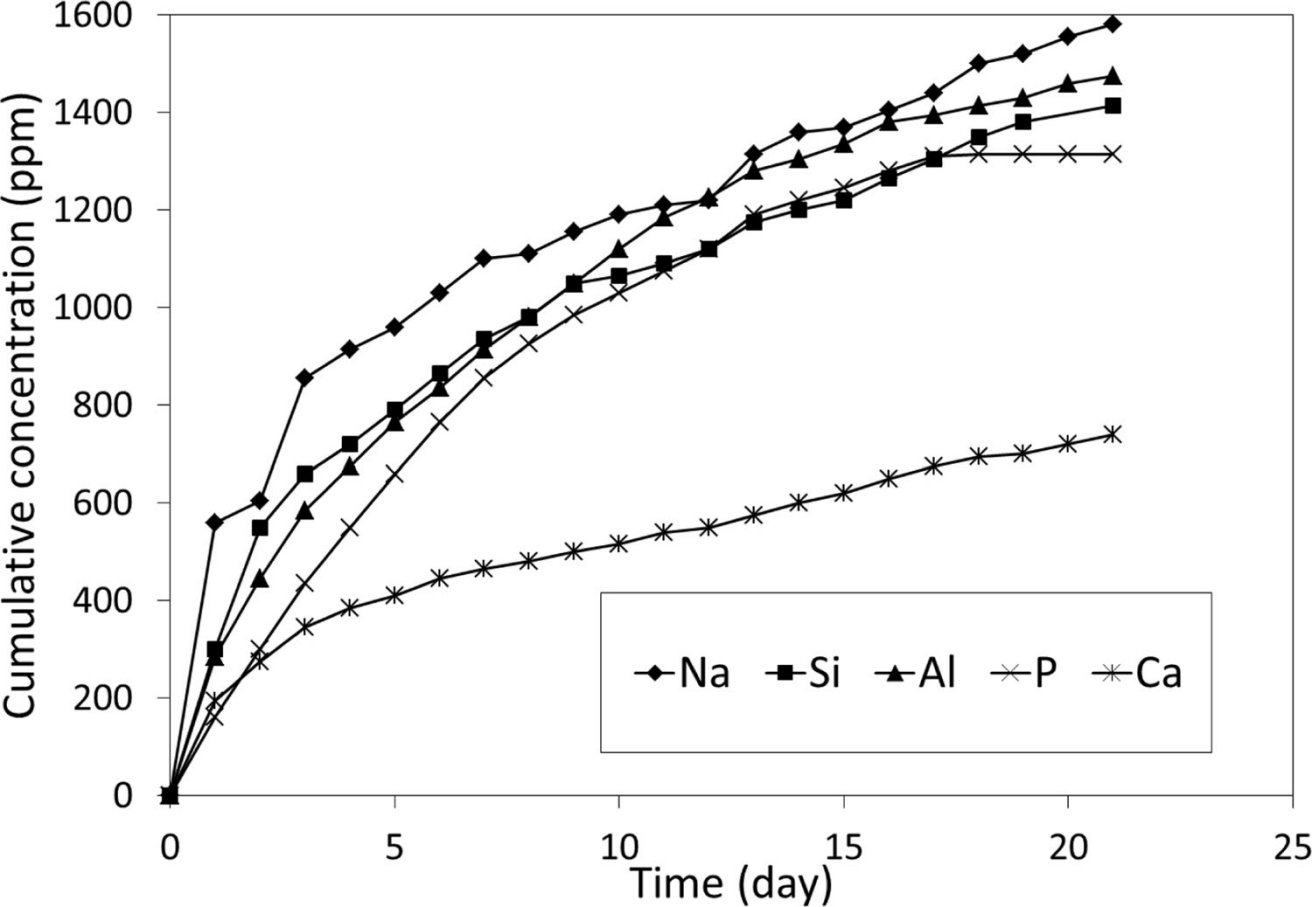
Twenty-one-month cumulative release of Na, Si, Al, P and Ca under acidic conditions

The plots of cumulative release of sodium, silicon, aluminium, phosphorus and calcium for the longer-term (21 month) studies have been made and are shown in Figs. 3 and 4 for neutral and acidic conditions, respectively. Cumulative total releases are listed in Table 3. In the case of calcium and phosphorus, there was only negligible release in neutral conditions over 1-month timescales. By contrast, in all other cases, there was a reasonable ion release at these timescales. In all cases, there was greater release in acidic conditions than in neutral ones, with this difference being significant for all the elements to at least *p* < 0.01.

**Table 3:** Cumulative release totals for all ions in long-term (21 month) experiments in neutral and acidic conditions (standard deviations in parentheses)

Element	Neutral conditions (ppm)	Acidic conditions (ppm)
Na	1365 (39.9)	1582 (93.0)
Si	549 (10.5)	1413 (41.5)
Al	409 (16.8)	1475 (40.3)
P	18 (0.1)	1316 (60.8)
Ca	Negligible	738 (31.0)

The results of curve fitting to Eqs. 3 and 4 are shown in Table 4. From the *R*^2^ values, it was apparent that under neutral conditions Eq. 3 gave the better fit to the data for those elements that were released in reasonable amounts, i.e., Na, Si and Al. By contrast, under acid conditions, Eq. 4 generally gave the better fit. The exception was aluminium release in acidic conditions, where the *R*^2^ values were the same for both equations, which meant that it was not possible to choose between the two equations.

**Table 4:** *R*^2^ values of equations for cumulative monthly ion release over 21 months

Element	Equation	Neutral conditions	Acidic conditions
Na	3	0.997	0.995
	4	0.996	0.997
Si	3	0.996	0.998
	4	0.995	0.997
Al	3	0.998	0.998
	4	0.997	0.998
P	3	–	0.996
	4	–	0.972
Ca	3	–	0.998
	4	–	0.996

In all cases, the statistical tests applied are strictly applicable where the data are normally distributed only. We have not tested for normality because of the limited numbers of measurements per specimen (6). However, this is the usual way in which data of this type are treated. Where it has been tested, and we have no reason to assume that our data should not be normally distributed.

## Discussion

The particular brand of glass-ionomer cement has been shown to release sodium, silicon, aluminium and phosphorus under both neutral and acidic conditions, and calcium under acidic conditions, when stored at 20–22 °C. This temperature is below that encountered by the cements in service, but is acceptable for the aims of the present study, which was aimed at comparing values, rather than modelling in vitro release.

The initial study involved release into water only, with measurements made daily for 28 days. This was to allow comparison with previous studies of fluoride release, which used similar storage times in order to determine kinetic parameters for the release [10, 11]. For these experiments, values of *β* in the same units we have used, i.e., ppm h^−½^ work out as lying between 34 and 147, compared with our highest value of 325 ppm h^−½^ for sodium and lowest of 3.5 ppm h^−½^ for phosphorus. In the previous studies, values of *t*_½_ varied widely, depending on the brand of glass-ionomer, and ranged from 8.5 to 57.8 h. Values around 24 h or just under were found most frequently, suggesting that this is a typical figure. In our studies, values of *t*_½_ for the ions ranged from 12.7 (Si) to 18.6 (P), in other words, were of the same order as those previously found for fluoride. Also, since simple calculation shows that it takes seven half-lives for release to be more or less finished, complete release of these ions can be seen to take between 3.5 and 6 days, depending on the element.

Longer-term experiments involved monthly measurements for 21 months. Using longer-term individual storage periods alters the amount of ion released, as has been shown previously [2], but allows longer-term kinetic behaviour to be determined. These experiments involved storage in neutral and acidic conditions and showed that, for all ions that were released in neutral conditions, considerably greater amounts were released into acid solution. This confirms previous observations [10, 11, 14, 15]. Where there was sufficient dissolution, i.e., for sodium, aluminium and silicon, release was found to follow Eq. 3 in neutral conditions. In acidic conditions, by contrast, release followed Eq. 4, though in the case of aluminium, Eq. 4 did not fit the data any better than Eq. 3. Both of these behaviours mimic that of fluoride release from glass-ionomer cements. In both conditions, these kinetic equations demonstrate that release occurs by two-step processes for all ions.

Previous studies on fluoride release have shown that the amount released is influenced by the frequency with which the storage medium is changed [28]. Another study measured fluoride release at daily intervals, changing the storage water only after 4 days, and when the water was refreshed in this way, there was a spike in the release [29]. The reasons for this are not entirely clear. It may be that the solvating ability of the water is better for pure water than for water that contains dissolved ions. However, the amount that dissolves is small, so for this to have a significant effect of solvating ability is surprising. Alternatively, there may be an equilibrium established between the ions in solution and the ions in the cement, so that the amount measured is the result of the balance between fluoride released and fluoride taken up. If this is the case, changing the water might be expected to shift the equilibrium in such a way that the overall amount of fluoride released increases.

This is particularly relevant in the present study in the case of release of phosphorus. In the short storage time experiments with daily changes of water, sufficient phosphorus was released to allow the release parameters *t*_½_ and *β* to be determined. However, for the long-term storage experiment, with monthly changes of the storage water, release was so low that there was not enough to compare with that predicted by either of the equations. This suggests that there is a change in the solubility of the phosphorus species with ageing, a change that is consistent with previous studies showed that slow changes in the nature of the phosphorus species occur as cements mature [30].

For fluoride release, the value of *β* has been recognized as a measure of the driving force for release. In the present study, this may be the same. Sodium shows the highest value, and also the largest amount released, which is consistent with it having the largest driving force for release (Table 2). By contrast, phosphorus has both the lowest value of *β* and the smallest release, suggesting that it has the smallest driving force for release of all the ions studied.

The release of these ions, and their dependence on the pH of the surrounding aqueous medium, shows that dissolution of glass-ionomers is incongruent, i.e., the dissolved material has a different composition from that of the solid. The part of the difference arises from the insolubility of the calcium component, which appears related to the low solubility of the phosphorus species.

The previous work has considered the nature of this species, and shown it to be mainly orthophosphate and possibly mono-fluorophosphate [17]. In the presence of calcium, there is the strong possibility of the formation of at least one type of discrete calcium phosphate compound. There are 11 known distinct calcium phosphates [31], and their solubility in water varies widely, depending on the Ca:P ratio. Low values of this ratio, i.e., 0.5 or slightly higher, give soluble compounds, whereas higher values, notably 1.67, give very insoluble substances. An indication of the insolubility is the solubility product, which is defined as a specific equilibrium constant for the process:

$${\mathrm{Ca}}_{\mathrm{5}}\left( {{\mathrm{PO4}}} \right)_{\mathrm{3}}\left( {{\mathrm{OH}}} \right) \rightleftharpoons {\mathrm{5Ca}}^{\mathrm{2 +}} + {\mathrm{3PO4}}^{{\mathrm{3 - }}} + {\mathrm{OH}}^{\mathrm{ - }}$$

i.e., *K*_sp_ = [Ca^2+^]^5^[PO_4_^3−^]^3^[OH^−^]

The values ranging from 10^−55^ [32] to 10^−62^ [33, 34] have been suggested for this constant under neutral conditions, and these extremely low values in turn imply that there are very low concentrations of the dissolved ions relative to the amount of insoluble substance present. In other words, the compounds are extremely insoluble. The very low amounts of phosphorus found in the solutions in the present experiments, particularly those changed each month rather than daily, suggest that a phosphate with a high Ca:P ratio is formed within the cement, and this ensures that both the calcium and phosphate hardly dissolve at all. By contrast, in acid solution, the solubility of high Ca:P ratio calcium phosphates is much greater [31], and this is reflected in the high calcium and phosphorus concentrations observed.

Unlike phosphorus, so far there has been no work to determine the species of silicon that is released, though at least one group describe it is Si^4+^ [16], which is obviously incorrect. The speciation of silicon is not straightforward, as can be seen by considering results from other relevant studies.

First, with the glass from which glass-ionomer cements are made, silicon is known to occur in the form of SiO_4_^4−^ tetrahedra. These tetrahedra link to three or four aluminium atoms via Si–O–Al bonds [35]. Aluminium is typically found in 4-coordination, i.e., in AlO_4_^5−^ tetrahedra, a structure forced on it by the presence of the large amounts of SiO_4_^4−^. Glasses with high fluoride content also contain reasonable amounts of aluminium in 6-coordination [36]. Even with high fluoride content, glasses show no evidence of any direct Si–F bonding [35]. When glasses dissolve following acid attack, transfer of hydrated silicate species out into the surroundings would be expected to occur, with them either existing as individual hydrated tetrahedra or as part of larger condensed species that the surrounding aluminate tetrahedra. The details of how this transfer might occur has not been studied.

By contrast, the nature of the silicate species that can form in aqueous solution has been studied quite extensively. Various silicates have been dissolved in water [37], including so-called water glass, a simple glassy substance with the formula Na_2_O.*n*SiO_3_ (*n* = 1–3.35) that dissolves readily and gives rise to numerous possible silicate species [38, 39]. Most of these are able to co-exist with each other under any given set of experimental conditions.

The species have been studied with a variety of techniques, including ^29^Si NMR and vibrational spectroscopy (FTIR and Raman). Using these techniques, species have been observed with varying degrees of polymerization, typically dimers to tetramers [38], with no observable monomers [38, 39]. Cyclic species have also been observed [37]. The degree of polymerization appears to be controlled by the pH and the concentration of Na^+^, and most conditions lead to the occurrence of reasonable amounts of trimers and tetramers in solution [40]. How this type of polymerization might be influenced by the presence of substantial amounts of AlO_4_^5−^ tetrahedra is not clear. The structural similarity with SiO_4_^4−^, and also the similar nature of the counter-ions, notably Na^+^, suggests that silicon may well be released from glass-ionomers as a series of pure silicate and alumino-silicate oligomers.

Similarly, the aqueous chemistry of aluminium is complicated, and the species of aluminium that might be released from glass-ionomers is not immediately obvious. In aqueous solutions of simple salts, such as halides or nitrates, several aluminium species have been identified, such as Al(H_2_O)_6_^3+^, AlOH(H_2_O)_5_^2+^, Al(OH)_2_(H_2_O)^4+^ and Al(OH)_4_(H_2_O)^4–^ [41–43]. As well as these monomeric species, several polymerized species have been identified, mainly dimers and trimers. Given that aluminium eluted from a glass-ionomer began inside the glass as AlO_4_^5−^ tetrahedra or similar species with direct Al–F bonds [40], it is unlikely that the main species in solution is the simple hydrated Al^3+^ ion. Rather, it seems likely that aluminium occurs in some sort of complex species that includes direct Al–O covalent bonds of the type that were present in the glass. As already mentioned, the variety of aluminium species in aqueous solution may also include alumino-silicate oligomers. It may well be that the relatively large size of these species is the reason for the slow and relatively limited amount of aluminium released by the cements, and may be the reason for the relatively small value of *β* for aluminium in Eq. 3.

## Conclusions

This study has shown that ions of the elements sodium, silicon, aluminium and phosphorus behave similarly to fluoride with respect to their release kinetics from a conventional glass-ionomer cement. In neutral conditions, release occurs by a two-step mechanism. Step 1, early washout, is rapid and characterized by half lives in the range 12–18 h, and completion times of 3.5–6 days. Step 2 is longer term and diffusion based.

In acidic conditions, there is much greater total release, and also elution of calcium, which was not observed in neutral conditions. Like fluoride, kinetics for all these elements change and become described by a two-step mechanism comprising early washout followed by longer-term erosion.

Possible species formed by silicon and aluminium in aqueous solution are discussed, and shown to be complicated, involving polymerization and, in the case of silicon, possibly cyclization as well. Phosphorus has previously been shown to form only two species on release, with PO_4_^3−^ being the predominant one. These differences in structure and in release with external pH are important in understanding the bioactivity of conventional glass-ionomer cements.
